# Infrared Spectroscopy of Neutral and Cationic Sumanene (C_21_H_12_ & C_21_H_12_^+^) in the Gas Phase: Implications for Interstellar Aromatic Infrared Bands (AIBs)

**DOI:** 10.1021/acsearthspacechem.4c00393

**Published:** 2025-03-27

**Authors:** Pavithraa Sundararajan, Piero Ferrari, Sandra Brünken, Wybren Jan Buma, Alessandra Candian, Alexander Tielens

**Affiliations:** † Leiden Observatory, Leiden University, Einsteinweg 55, 2333 CC Leiden, The Netherlands; ‡ Institute for Molecules and Materials, FELIX Laboratory, Radboud University, Toernooiveld 7, 6525 ED Nijmegen, The Netherlands; § Anton Pannekoek Institute, University of Amsterdam, Science Park 904, 1098XH Amsterdam, The Netherlands; ∥ Astronomy Department, University of Maryland, College Park, Maryland 20742, United States

**Keywords:** interstellar chemistry, infrared spectroscopy, organic molecules, aromatic infrared bands, polycyclic aromatic hydrocarbons, buckybowls

## Abstract

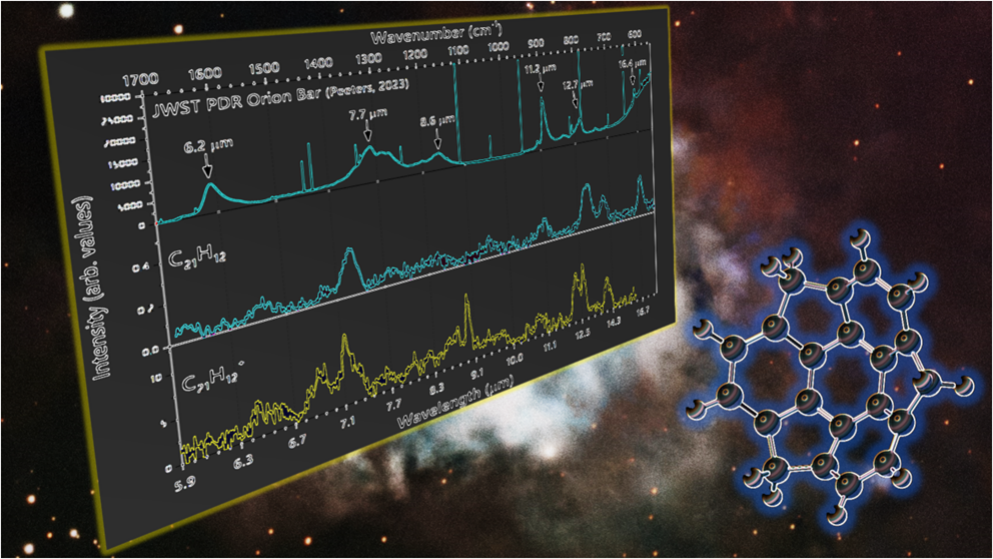

Polycyclic aromatic hydrocarbons (PAHs) are known to be omnipresent in various astronomical sources. Ever since the discovery of C_60_ and C_70_ fullerenes in a young planetary nebula in 2010, uncovering the reaction pathways between PAHs and fullerenes has been one of the primary goals in astrochemistry. Several laboratory studies have attempted to elucidate these pathways through experiments simulating top-down and bottom-up chemistry. Recently, indene (c-C_9_H_8_, a fused pentagon and hexagonal ring) has been detected in the TMC-1 molecular cloud. This is a significant finding since pentagon-bearing PAHs could be key intermediates in the formation of fullerenes in space. Spectroscopic studies of pentagon-bearing PAHs are thus essential for their detection in molecular clouds, which would eventually lead to unraveling the intermediate steps in PAH’s chemistry. This work reports the infrared (IR) spectra of both neutral and cationic sumanene (C_21_H_12_ and C_21_H_12_^+^): a bowl-shaped PAH containing three pentagon rings. Apart from its relevance for furthering our understanding of the chemistry of PAHs in an astronomical context, the presence of three sp^3^ hybridized carbons makes the vibrational spectroscopy of this molecule highly interesting also from a spectroscopic point of view, especially in the CH stretching region. The experimental IR spectra of both species are compared with quantum chemically calculated IR spectra as well as with the aromatic infrared bands (AIBs) of the photodissociation regions of the Orion Bar obtained using the James Webb Space Telescope (JWST).

## 1 Introduction

The cosmic electromagnetic spectrum in the visible, electronic, and submillimeter regions is dominated by molecular absorptions and emissions bands.^1^ The infrared (IR) region is of utmost interest to astrochemists, as it reveals the vibrational molecular fingerprints. The aromatic infrared bands (AIBs) are discrete infrared emission bands observed in the interstellar medium (ISM), circumstellar regions, and galactic and extragalactic sources, with features at 3.3, 6.2, 7.7, 8.6, 11.2, 12.7, and 16.4 μm. By now it has been commonly accepted that they are associated with polycyclic aromatic hydrocarbons (PAHs),^2^ arising from IR emissions of UV-pumped PAH-type molecules that after internal conversion and intramolecular vibrational energy distribution relax through vibrational emission. PAHs are thought to be present in the ISM in different forms, including neutral, cationic, dicationic, protonated, hydrogenated, or dehydrogenated species.^3^ Despite many theoretical and experimental studies that are extensively documented in the NASA Ames PAH database (www.astrochemistry.org/pahdb),^4^ it still has not been possible to come to a specific assignment to individual PAHs or groups of PAHs responsible for the AIBs. In particular cases, the identification is possible with radio astronomy, and this has recently been demonstrated for the case of cyanopyrene (C_17_H_9_N).^5^ However, the recent infrared data obtained with the James Webb Space Telescope (JWST)—which provides significantly improved spatial and spectral resolution compared to the previous infrared space telescopes—is promising for the solution of this conundrum.^6^

Above and beyond fully benzenoid PAHs, the buckyball C_60_ has also been identified in the ISM by its strong vibrational transitions at 7.0, 8.6, 17.4, and 18.9 μm using observations from the Spitzer Space Telescope.^7^ Moreover, C_60_^+^ was also identified, through its electronic transitions.^8^ The upper limit abundance of C_60_^+^ in NGC 7023 has been estimated to be <0.26% of the interstellar carbon abundance, whereas for neutral C_60_, the upper limit is <0.27%.^9^ Berné et al. gives 0.017% of the elemental carbon in neutral C_60_ in NGC 7023.^10^ Berne et al.^11^ gives 0.007% of the elemental carbon in C_60_^+^ and a C_60_^+^/C_60_ ratio of 0.38. This indicates the possibility of an ionized population of curved large hydrocarbons in interstellar clouds. Astronomical observations have led to the conclusion that the abundance of C_60_ increases rapidly close to stars while the abundance of PAHs increases away from them,^12^ likely reflecting photochemical fragmentation and isomerization processes under the influence of the strong radiation fields.^13^ Laboratory studies support the importance of UV-mediated reactions for the processing of fullerenes and cages from PAHs.^14^ This process might not be the only one at play: recent modeling work by Sidhu et al. has shown that the decrease in PAH abundance and the concomitant increase of C_60_ might be a geometric effect (geometric patterns in a nebula, primarily caused by the stellar winds and radiation from the central star interacting with the surrounding gas and dust) in that nebula.^15^

C_60_ has a cage-like structure consisting of fused benzene and cyclopentadiene rings, with pentagons leading to a curvature of the planar PAH structure closing upon itself.^16−18^ Pentagon formation is thus a prerequisite for the photochemical transformation of PAHs into fullerenes. Laboratory spectroscopy suggests that upon PAH photodissociation leading to the pentagon formation is quite pronounced, even when a carbon site is replaced with a nitrogen (PAHN).^19−22^ Besides this *top-down* chemical link between PAHs and fullerenes, the recent discovery of the fused pentagon-hexagon species indene (C_9_H_8_) in dark molecular cloud cores suggests that there may also be *bottom-up* routes from PAHs to fullerenes.^23−25^ These routes may be analogous to early laboratory studies on the formation of fullerenes.^26−28^ In either scenario, top-down or bottom-up, the recording and characterization of IR absorption spectra of stable pentagon-hexagon-bearing species is of prime importance for a search of their presence in space. The recent search for the buckybowls in the Red Rectangle nebula failed, and the quest still continues.^25^ Both rotational and infrared spectroscopy are critical for an unambiguous detection.

**Figure 1:**
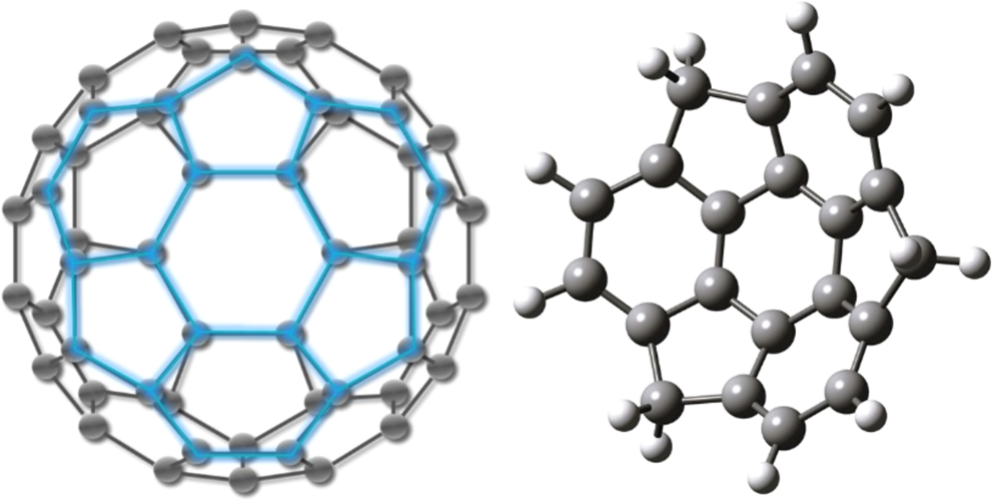
Sumanene (C_21_H_12_), a hydrogenated fragment of C_60_. The left image is the C_60_ molecule highlighting the C_21_ structural motif, which represents sumanene.

Sumanene (C_21_H_12_,) can be considered as one of the building blocks of C_60_ as shown in Figure 1. This bowl-shaped species that contains three peripheral pentagons may represent an intermediate in the formation routes of C_60_ in space.^29−31^ The photochemical evolution of sumanene has recently been studied in detail experimentally, while relevant fragmentation routes have been characterized using density functional theory (DFT) studies.^32^ Matrix-isolation spectroscopy in combination with DFT calculations was employed to study the IR spectrum of sumanene. These studies revealed four fundamental CH stretching modes along with intense CH *out-of-plane* bending modes.^33^ The prominent IR features corresponding to the CH stretching region of the AIBs are believed to be due to the aromatic CH stretching (3.3 μm) and aliphatic CH stretching (3.4–3.5 μm).^34^*The presence of three sp*^3^*-hybridized benzylic sites*—*a unique characteristic of sumanene*—makes the infrared spectroscopy of sumanene very interesting to study under astronomically relevant conditions, i.e., in the gas phase at low temperatures.^35^ Such studies are still notoriously missing yet essential for any astronomical detection under interstellar conditions. In this work, the infrared absorption spectra of neutral sumanene (C_21_H_12_) in the 3200–400 cm^–1^ region and cationic sumanene (C_21_H_12_^+^) in the 1600–400 cm^–1^ range are measured. Their implications and correlation with the mid-infrared spectra observed with the JWST are discussed with a focus on the CH stretching region (3–4 μm).

## 2 Experiment

IR absorption spectra have been recorded using the FELIX (Free Electron Laser for Infrared eXperiments)^36^ facility which provides continuous tuning of the wavelength over the 2.8–100 μm range, combined with high power and spectral resolution. Two different state-of-the-art experimental setups integrated as end user stations of the FELIX beamline, which will be discussed in more detail below, have been used for this work. Experiments have been performed on sumanene powder purchased from Tokyo Chemical Industry (C_21_H_12_, 99.0% pure).

### 2.1 Laser Desorption Molecular Beam

The mid-infrared spectrum of neutral sumanene was recorded with the laser desorption molecular beam setup at FELIX.^37−40^ To form a molecular beam of neutral sumanene, the sample was mixed with carbon black powder in a 1:1 ratio and pressed onto the surface of a solid graphite bar. Sumanene was brought into the gas phase using a pulsed 1064 nm Nd:YAG laser beam with a typical pulse energy of 1 mJ/pulse. The desorbed molecules were entrained in argon (backing pressure of 4 bar) and expanded into a vacuum with a pulse valve. During supersonic expansion, the molecules cooled and were subsequently passed through a 2 mm skimmer, resulting in intact, cold, neutral molecules. After formation and collimation, the molecular beam entered the ionization region of a reflectron time-of-flight mass spectrometer, where ions were perpendicularly extracted. At the entrance of the mass spectrometer, the beam was ionized using the light of a Xe/Ar cell (10.5 eV) generated from the third harmonic of a Nd:YAG laser (355 nm).

To record an infrared spectrum, the technique of infrared multiple photon dissociation (IRMPD) spectroscopy was employed.^41^ For this, the molecular beam was merged with the counterpropagating light of FELIX 300 μs prior to ionization. Given the high power of FELIX and its pulse structure of roughly 10 μs long macro pulses, multiple photons are absorbed if excitation occurs in resonance with a vibrational mode. The gained energy is then rapidly redistributed via infrared vibrational redistribution (IVR), until the dissociation threshold is surpassed. This leads to a depletion in the signal of the molecular ion and an ingrowth of the signal of masses associated with dissociation channels. In the case of sumanene, this was primarily the loss of one hydrogen atom. For the measurements, FELIX was operated at 5 Hz, whereas the molecular beam was pulsed at 10 Hz, which allowed recording consecutive mass spectra without and with FELIX excitation. FELIX was scanned in the range from 3200 to 400 cm^–1^ (3.125–25 μm) in steps of 2 cm^–1^. Each mass spectrum was an average of 50 shots at the same FELIX wavelength. The obtained final depletion IR spectra were then corrected using three steps: baseline correction, power correction, and wavelength calibration.

In addition to the experiments performed with FELIX, the range of 2850–3100 cm^–1^ was reexamined using a table-top infrared OPO/OPA laser (LaserVision). The line width of FELIX depends on the wavelength, being about 0.4% of the central wavenumber in these experiments. Therefore, the line width around 3000 cm^–1^ is close to 12 cm^–1^. Instead, the OPO/OPA laser has a fixed line width below 1 cm^–1^, allowing for spectra with a higher resolution. For these experiments, the technique of ion-dip spectroscopy was employed. For this, ionization occurs via a 1 + 1 REMPI scheme,^42^ with excitation taking place at 267 nm. Here, the skimmed sumanene molecular beam was crossed by the laser light of a dye laser operating on Coumarin 153 dissolved in ethanol and pumped by the third harmonic of a Nd:YAG laser. Typical pulse energies of around 1 mJ after doubling the fundamental light of the dye laser were employed.

### 2.2 22-Pole 4 K Cold Trap (FELion)

The infrared spectrum of the sumanene cation in the gas phase was recorded for the first time. In these experiments, cations were stored in the 22-pole cryogenic ion trap of the FELion instrument, and the IR spectra were obtained using the intense and tunable free electron lasers at the FELIX facility. The FELion 22-pole cryogenic ion trap instrument at FELIX has shown to be ideally suited to study PAH^+^ using IRPD (infrared predissociation) spectroscopy at a temperature as low as 4 K.^43^ A detailed description of this instrument along with its schematics has been given in previous work.^44^ IR-PD is an action spectroscopy method that uses the changes in the mass-to-charge ratio of the measured ions due to photon absorption, i.e., the loss of the tag atom. As such, the technique allows for the recording of experimental IR spectra that are not affected by anharmonic distortions and closely resemble the linear absorption spectrum of the bare ion, with respect to band positions and intensities. This technique enables the structural characterization of molecular ions that are otherwise hard to measure such as reactive species or small molecular ions for which infrared multiphoton dissociation (IRMPD) spectroscopy is not suitable. Recently, it has been used to record IR spectra of PAH^+^ to derive dynamical information successfully.^45,46^

In the present study, solid sumanene was loaded into the sample compartment and heated externally using heating tapes to 110 °C to bring it to the gas phase. The molecules were then transferred to the ion source by flowing helium gas through the sample compartment. There, sumanene was ionized by electron impact ionization using 50 eV electrons. The ionized molecules were extracted from the source in a few 10 ms long pulse and then selected for *m*/*z* 264 (sumanene cation) by using a quadrupole mass filter. They were then guided into the 22-pole ion trap that was kept at 6.8 K. To thermalize the ions and to form Ne–C_21_H_12_^+^ complexes through intermolecular collisions an ∼100 ms long gas pulse containing a 1:1 mixture of Ne/He was admitted into the trap around 15 ms before the ions reached the trap. The bond of PAH^+^ with a noble gas atom through very weak van der Waals forces is easily fragmented by IR one-photon absorption.

To record IR-PD spectra, the FELIX beam was steered into the cold trap and the Ne–C_21_H_12_^+^ complexes were mass-selected by the second quadrupole mass filter after the interaction time and detected with the Daly detector. The IR-PD spectrum was recorded by counting the Ne–C_21_H_12_^+^ ions as a function of wavelength over the range of 600–1800 cm^–1^. At each wavelength 16 macro pulses of FEL-2 with pulse energies of around 67 mJ were used to irradiate the ions The final spectrum had a typical bandwidth of (fwhm) Δν = 20 cm^–1^, and the bandwidth of the radiation is Fourier-transform limited and on the order of 0.6% of the center frequency. To obtain the full spectrum, data normalization was carried out as mentioned in the earlier work,^44^ i.e., normalization per photon to account for varying laser energy and number of ions. Three full-range spectra were averaged with a binning size of 3 cm^–1^. The wavelength was calibrated with a grating spectrum analyzer, leading to a typical uncertainty of the derived band positions of 1–2 cm^–1^.^47^

## 3 Theory

Neutral sumanene (C_21_H_12_) has 92 normal modes and belongs to the *C*_3*v*_ point group, with a 1A^1^ nondegenerate electronic ground state.^41^ Unlike C_60_, which suffers from Jahn–Teller distortion upon ionization because of its 5-fold degenerate electronic ground state, the radical cation belongs to the same point group as the neutral, also with a 1A^1^ electronic ground state.^11,16,48^ The geometry of neutral and cationic sumanene were optimized at the B3PW91/6-311++G(2d,2p) level of theory and harmonic calculations performed at this level, which has shown to produce vibrational spectra of medium-sized puckered PAHs in excellent agreement with experimental spectra.^30,49,50^ The harmonic frequencies ν_harm_ of neutral sumanene were scaled by 1.130.ν_harm_ – 526 and 0.982.ν_harm_ – 6 for >2000 and <2000 cm^–1^, respectively, to account for the difference in the degree of anharmonicity between the CH stretching and lower-frequency modes as determined by Weber et al. (2022).^33^ The harmonic vibrational frequencies of the sumanene cation were scaled with factors of y = 0.9807.ν_harm_ for <2500 cm^–1^. In addition, anharmonic calculations using generalized second-order vibrational perturbation theory (GVPT2) implemented in the GAUSSIAN 16, (version C.02)^45^ were performed, using the B3LYP/N07D level of theory which has shown to provide a good compromise between accuracy and computational time in previous studies of PAHs.^46,51,52^ The anharmonic spectrum of cationic sumanene (optimized with a structure belonging to the C_1_ point group) was obtained using default settings in the Gaussian input except for: (1) using a threshold of 100 cm^–1^ for Fermi and Darling-Dennison resonances and (2) freezing the low-frequency normal modes 93, 92 and 90. These restrictions significantly improved the appearance of the anharmonic spectrum, removing unrealistically high infrared intensities and large negative anharmonic corrections to modes frequencies. In the case of neutral sumanene, these restrictions did not improve the appearance of the anharmonic spectrum. Use of a different basis set with the B3LYP functional did not improve the situation, and we suspect that the origin of the problem is the low quality of the QFF. We decided to not include the anharmonic calculation for neutral sumanene in this paper and investigate the issue further. In the remainder of this paper, we rely on the scaling factors to infer the anharmonic corrections. The calculated harmonic and anharmonic vibrational frequencies and IR intensities of neutral and cationic sumanene are listed in Tables S1–S3.

## 4 Results and Discussion

### 4.1 Infrared Spectra of Sumanene (C_21_H_12_)

**Figure 2:**
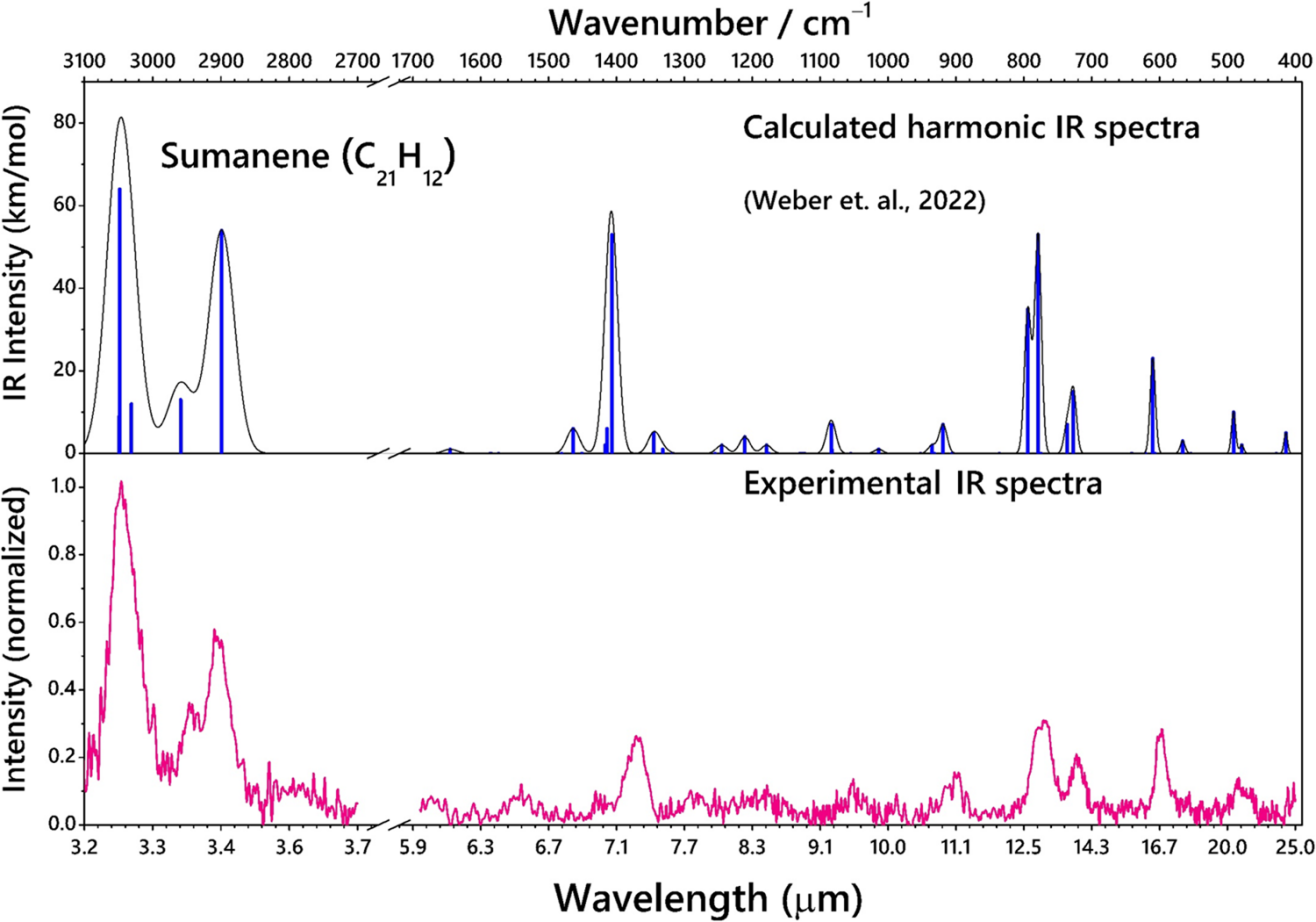
IR absorption spectra of neutral sumanene in a molecular beam (bottom; pink), together with predicted spectra using the harmonic approximation with scaled frequencies (top; blue).^28^ The black trace is a convolution of the stick spectrum with the FELIX line shape.

The IR absorption spectrum of jet-cooled neutral sumanene in the 3–100 μm (FELIX, 100–3200 cm^–1^) region was measured using the laser desorption molecular beam method as shown in Figure 2. Since the width of the peaks in the FELIX region is determined by the bandwidth of FELIX, the line widths of the IR bands are narrower in the far-IR than in the mid-IR region.

Figure 2 compares the experimentally recorded IR spectrum of sumanene with the predicted IR harmonic calculations (B3PW91 6-311++G(2d,2p) method).^28^ The theoretical predictions are in good agreement with the experimental spectra. The experimental spectrum displays the most intense bands in the FELIX region associated with the CH out-of-plane (CH_oop_) modes of duos and CH_2_ groups, respectively, at approximately 770 (±2) and 722 (±2) cm^–1^. Both the bands are strikingly different from what is observed for corannulene (C_20_H_10_), another buckybowl, which contains only sp^2^ C bonds in the periphery and only one intense feature of the CH_oop_ band at 837 cm^–1^.^48^ The CC stretching mode is predicted at 1371 cm^–1^ and is predicted at 1407 cm^–1^ by using the harmonic approximation. The intense CH stretching modes corresponding to the sp^2^ and sp^3^ hybridized sites are observed at 3047 (±2) cm^–1^ and 2905 (±2) cm^–1^; and the predicted modes at 3060 and 2934 cm^–1^, respectively. Several CH_2_ stretching and wagging modes are visible below 700 cm^–1^, among which the two intense CH_2_ rocking modes are observed at 596 (±2) cm^–1^ and 483 (±2) cm^–1^; and the ring breathing mode at 556 (±2) cm^–1^. Like the case of corannulene,^41^ the CC stretching mode (1400–1600 cm^–1^) of sumanene is very weak. This weakness is in contrast to the moderately intense peaks due to CC modes in neutral PAH of similar size (e.g., coronene, pyrene), and the cause of this is the planarity.^53^ Because of the presence of both sp^2^ and sp^3^ hybridized C bonds, the CH_oop_ modes seem to combine with the other combination modes to give rise to several moderately intense peaks in the 1000–400 cm^–1^ region.

**Figure 3:**
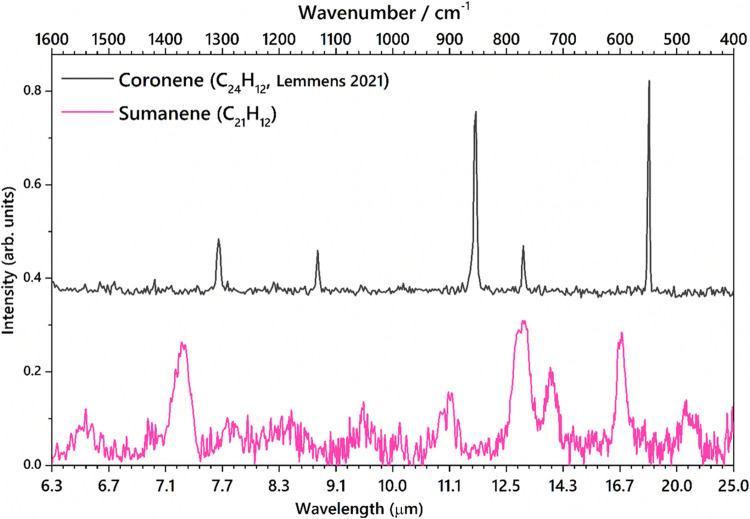
Comparison of the IR absorption spectra of sumanene (bottom; pink (this work)) with that of coronene (top; black (data obtained from Lemmens et al., 2021)).^54^ Both spectra are measured in a molecular beam using the same experimental approach.

Figure 3 shows a comparison of the IR spectra of sumanene and coronene in the gas phase, recorded with the same molecular beam system. The 1600–400 cm^–1^ regions also known as the fingerprint region harbor several motions concerned with the CH in-plane, out-of-plane, and the CC bonds that need to be compared between sumanene and coronene to understand the influence of nonplanarity in the IR spectrum. The IR bands of coronene are narrower compared to those of sumanene, likely for two reasons: the higher number of IR active modes for sumanene (see Figure 2) and the line width of FELIX at the time of the experiments. The 850–1300 cm^–1^ region in sumanene is dominated by several weaker modes including CH in-plane bending modes. The CH in-plane bending modes of sumanene are blue-shifted from that of coronene, which could be due to the curvature of sumanene. These modes are not observed in coronene given the presence of only sp^2^ hybridized carbon and its compact structure. The CCC skeletal mode of coronene is at 549 cm^–1^, which is blue-shifted to 597 cm^–1^ in sumanene. Moreover, the CH_oop_ bending mode of coronene is at 854 cm^–1^, which is red-shifted to 770 cm^–1^ in sumanene. The CC stretching mode of sumanene at 1370 cm^–1^ is much more intense compared to that of coronene at 1306 cm^–1^. There are two distinct features that are exclusively observed in sumanene only because of the sp^3^ hybridized H atoms – (i) the two distinct peaks of the CH_oop_ bending modes and, (ii) the moderately intense peak at 897 cm^–1^ which is the combination of CH_oop_ bending mode of the sp^3^ group along with the C_2_H_2_ twisting mode of the duo CH groups attached to the hexagons. This comparison clearly depicts the influence of the curvature induced by pentagon rings on the IR spectrum.

**Figure 4:**
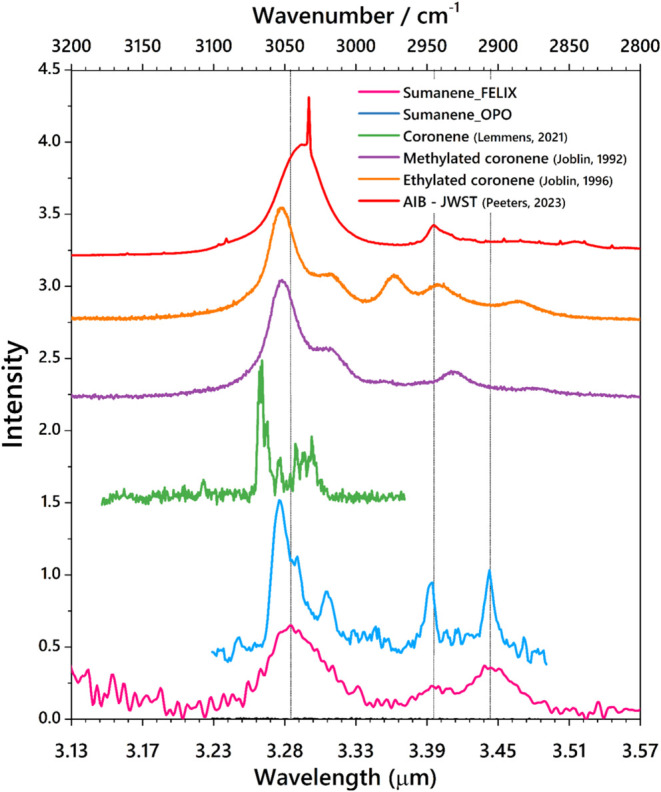
IR absorption spectra of neutral sumanene in the molecular beam measured with FELIX (pink, this work) and with the OPO/OPA table-top laser (blue, this work). The figure also presents the IR spectrum of coronene in a molecular beam (green; data obtained from Lemmens et al., 2021),^53^ methylated coronene (violet; obtained from Joblin et al., 1995)^55^ and ethylated coronene (yellow; data obtained from Joblin et al.)^53^ in the gas phase, and the interstellar AIB in the photodissociation region (PDR), recorded by the JWST (red; data obtained from Peeters et al., 2024).^62^ The vertical lines indicate the exact peak positions of the CH stretching modes of sumanene.

Figure 4 compares the presently recorded IR absorption spectrum of neutral sumanene in the 3 μm region with the spectrum of coronene recorded in a molecular beam (Lemmens).^54^ In addition, the IR spectra of methylated coronene (Joblin, 1995)^55^ and ethylated coronene (Joblin, 1996),^53^ both measured in the gas phase, are shown. It should be noted that the IR spectrum of sumanene was recorded in a cold beam, leading to vibrational temperatures in the order of 50 K,^56−59^ whereas the methylated and ethylated coronene measurements were conducted at 450 °C (723 K).^44^ These spectra are compared with the JWST observations of the interstellar AIBs in the photodissociation region (PDR) of the Orion bar (Peeters, 2024).^60,62^ It is observed that the aromatic component gives rise to a single intense feature along with a shoulder in the 3.3 μm region, whereas the presence of aliphatic components heavily alters the 3.4 μm region.

In general, the CH stretching vibrations can help decipher the periphery of a PAH, especially between the sp^2^ and sp^3^ hybridized CH bonds. The characteristic CH stretching modes of sumanene lie at 3047 cm^–1^ (3.28 μm), which is slightly red-shifted from that of coronene, methylated coronene, and ethylated coronene present at 3065, 3052, and 3051 cm^–1^, respectively. In addition, sumanene, methylated coronene, and ethylated coronene show two unresolved bands around the 3.27–3.33 μm region, one being more intense. Coronene, on the other hand, shows many well-resolved bands in the 3025–3075 cm^–1^ region. The aliphatic groups attached to the parent PAH play a major role in the IR signatures of the 3000–2850 cm^–1^ (3.33–3.5 μm) region. The CH_2_ stretching mode of the three CH_2_ groups attached to the peripheral pentagons of sumanene is observed at 2905 cm^–1^ (3.44 μm). The asymmetric stretching mode of this CH_2_ group is present at 2946 cm^–1^ (3.39 μm). The theoretical simulations using DFT (Figure 2) show that all three CH_2_ stretching modes are equivalent. The CH_3_ aliphatic modes of methylated coronene are centered at 2931 cm^–1^ (3.41 μm). There are multiple aliphatic modes observed in the case of ethylated coronene at 2973 cm^–1^ (3.36 μm), 2940 cm^–1^ (3.40 μm) and 2885 cm^–1^ (3.46 μm). The intensity ratio of the aliphatic to aromatic bands of methylated coronene and ethylated coronene is estimated to be as large as ∼0.3, whereas the corresponding ratio is ∼0.5 for sumanene. Given the 1:1 ratio of aliphatic (CH_2_) to aromatic groups in sumanene, we can conclude that the intrinsic IR activity of stretches involving the CH_2_ group is lower than that of stretches involving the methyl CH_3_ group (a 1:6 ratio in methylated coronene).

Though the 3.3 μm region and 3.4 μm region are dominated by the aromatic and aliphatic modes, respectively, they are not purely aromatic and aliphatic modes according to a recent work by Esposito et al.^63^ The authors after studying the experimental and theoretical IR spectrum of indene and 2-ethenyltoluene inferred that these modes in the 3.3 and 3.4 μm regions are a mix of aliphatic and aromatic CH modes and, in certain cases, a mix of non-CH modes like CC stretches. Likewise, sumanene being a molecule that possesses both sp^2^ and sp^3^ CH motions, and because the anharmonic calculations provide a mix or a combination of CH/CH_2_/CC motions, it is not possible to disentangle the “pure” aliphatic and aromatic CH motions in this case. Hence, the terms aliphatic and aromatic mentioned in this work point only to the motion that has the “major” vibrational contribution.

The CH stretching features of sumanene are also different from the other methylated and (super)hydrogenated, PAHs. For instance, the IR spectrum of 9,10-dimethyl anthracene shows strong additional IR features compared to anthracene centered at around 2849 and 2760 cm^–1^.^52^ These aliphatic peaks are red-shifted from those of sumanene, by about 50–60 cm^–1^. Likewise, hexahydropyrene has several intense IR lines centered at 2950 cm^–1^ and extending up to 2800 cm^–1^, a region that is also red-shifted from what is observed for sumanene. An important reason for the appearance of numerous IR peaks is that the addition of a methyl side group or an additional hydrogen to a PAH lowers the symmetry of the molecule leading to more IR active modes in the CH stretching region and more chances of resonances. Neutral sumanene, on the other hand, has *C*_3*v*_ symmetry and, hence, fewer active modes in the CH stretching region. These observations on the positions and relative intensities of the aromatic bands and the aliphatic side groups attached to a PAH clearly emphasize that the 3 μm region can be used as a sensitive probe for its characteristics. The observed interstellar AIB spectrum characteristically shows IR emission over a very limited spectral range in the aromatic CH stretching region and both experiments and theory imply that the emission is carried by highly symmetric neutral species such as coronene or, as demonstrated here, sumanene.^38,53,57^ Laboratory measurements thus provide key input for the interpretation of astronomical observations and modeling studies that study the evolution of carbonaceous dust.

### 4.2 Infrared Sectra of Sumanene Cation (C_21_H_12_^+^)

**Figure 5:**
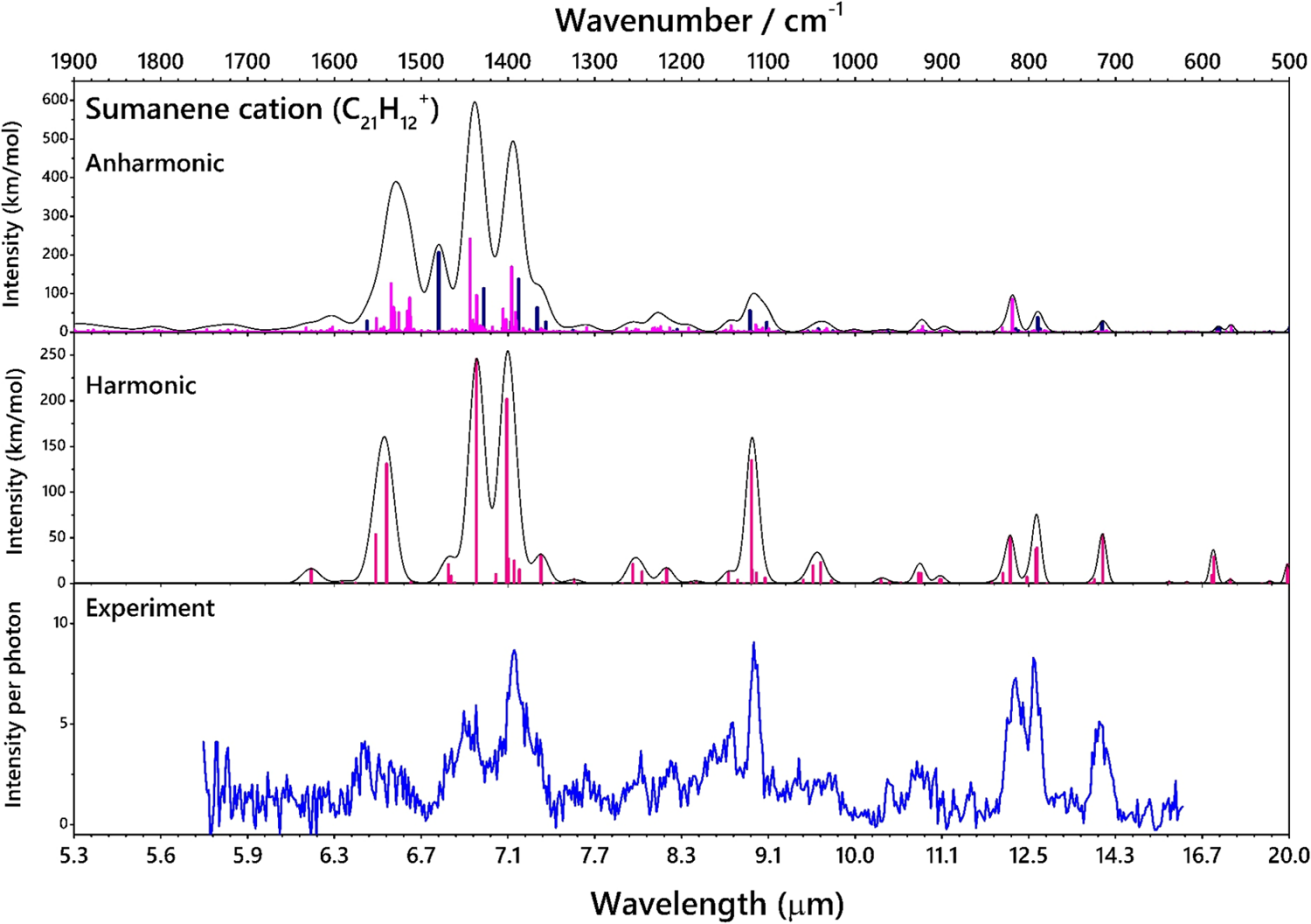
Experimental IR-PD spectra of Ne-tagged C_21_H_12_^+^ compared with the calculated scaled harmonic spectra of C_21_H_12_^+^ using the B3PW91 6-311++G(2d,2p) method and the anharmonic frequencies calculated with B3LYP/N07D method (blue lines—fundamental modes, pink lines—combination modes). The black trace is a convolution of the stick spectrum with a FELIX line shape.

The IR-PD spectrum of the Ne-tagged sumanene cation (C_21_H_12_^+^) compared with the predicted scaled harmonic IR spectrum is shown in Figure 5. The scaled harmonic spectrum provides an excellent correlation with the experimental spectrum of cationic sumanene but is not able to reproduce fully the wealth of minor features. The full list of frequencies and intensities is provided in Table S2. To reproduce the full wealth of the IR features, anharmonic calculations become necessary to provide information about the combination bands and overtones. In the anharmonic stick spectrum, the blue lines represent the fundamental modes and the pink lines are the combination bands. There are a total of 4095 combination modes predicted by theory. The list of anharmonic lines with normalized intensities >0.5 is provided in Table S3. The presence of combination bands and resonances results in a more complex spectrum, with several intense bands in the 1600–1350 cm^–1^ region. In these experiments, the Ne-tag is likely to induce small shifts, typically in the order of a few wavenumbers.^45^ Comparatively weak IR features were also observed and assigned. The observed typical shifts between the experimental and predicted harmonic IR frequencies were ∼14 cm^–1^, and the average shifts between the experimental and the anharmonic IR frequencies were 6.6 cm^–1^. There are two distinct CH_oop_ modes present here: (i) two CH_oop_ bending modes of sp^2^ and sp^3^ hybridized CH bonds predicted at 790.5 and 791.7 cm^–1^ which appears as a single feature in the experimental spectrum at 795 (±2) cm^–1^; (ii) a CH_oop_ bending mode of only the sp^2^ hybridized CH bond at a higher wavenumber observed experimentally at 815 (±1) cm^–1^. A strong CH_2_ wagging mode combined with a CCC skeletal mode is observed at 719 cm^–1^. There are several weaker CH_2_ rocking modes that are observed as a broad feature centered at 929 (±2) cm^–1^. The strong CH in-plane bending mode is observed at 1117 (±1) cm^–1^ (8.9 μm). The characteristic C–C stretching modes are observed at 1393 (±1) and 1437 (±1) cm^–1^. A few strong modes in the 1500–1600 cm^–1^ region, among them the CH in-plane bending modes, are observed as a plateau centered at 1565 (±1) and 1654 (±1) cm^–1^. These are likely to arise from combination modes.

**Figure 6:**
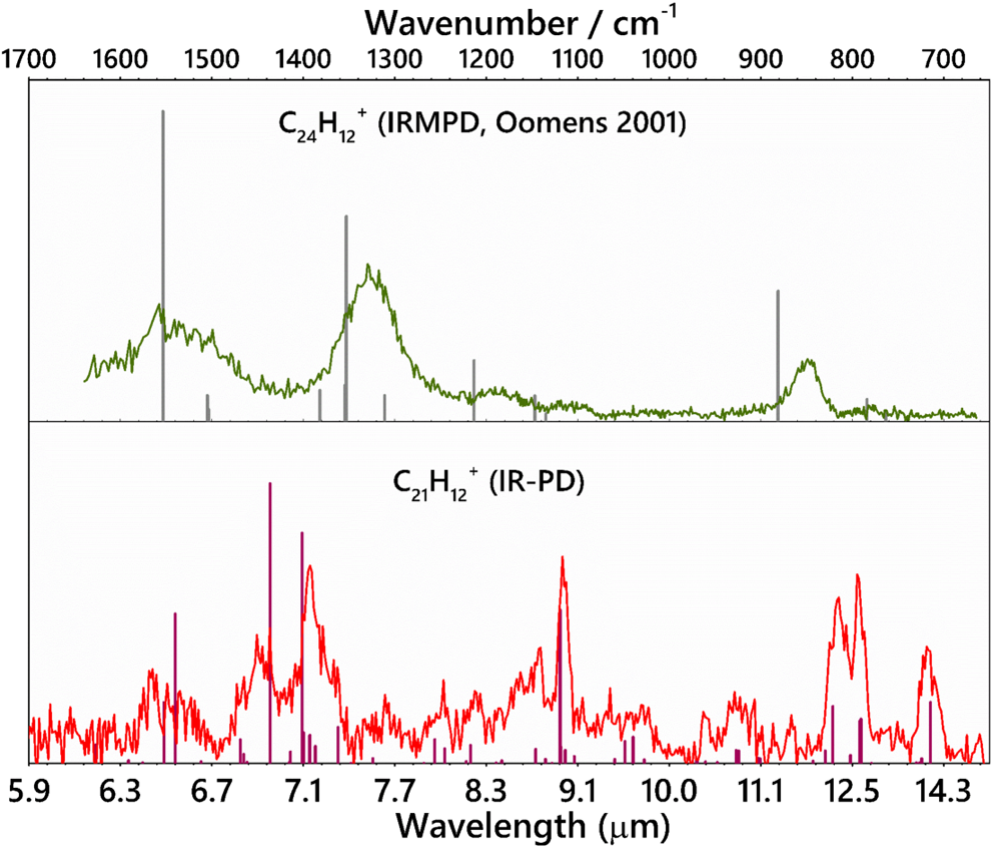
IRPD spectrum of the sumanene cation (C_21_H_12_^+^, this work) recorded in the cryogenic ion trap (red), compared with the room-temperature IR spectrum of the coronene cation (C_24_H_12_^+^) obtained by Oomens et al., with IRMPD (black) in the 700–1700 cm^–1^ regions.^30^ The stick spectra represent theoretically predicted harmonic frequencies.

To understand the influence of the planarity and the pentagon rings on the infrared signatures, we compare in Figure 6 the IR absorption spectrum of the bowl-shaped sumanene cation with that of the planar coronene cation. The increased IR activity of the CC modes in the puckered PAH, sumanene, as compared to the highly symmetric flat PAH, coronene, manifests itself through a broadening of the relevant modes. The major difference between sumanene and coronene cations lies in the relative intensities of the CH_oop_ and CC stretching modes. The CC stretching mode (1380 cm^–1^) is the most intense feature for the coronene cation, whereas both the CC and CH_oop_ modes are equally intense for cationic sumanene (1393 and 1437 cm^–1^). The fwhm of the CC stretching mode of sumanene (1393 cm^–1^) is ∼20 cm^–1^, whereas, for coronene (1380 cm^–1^), it is ∼35 cm^–1^. One main reason for the increased bandwidth of all of the bands in coronene lies in the fact that it is an IRMPD spectrum at room temperature. Hence, the bands are intrinsically broader.^30^ So, discussing planarity based on the widths is, in principle, not possible. Hence, this discussion will elaborate on the vibrational modes that are a consequence of this PAH’s lack of pure planarity. A striking difference between the two spectra is the CH in-plane bending mode at 1117 cm^–1^, which is almost absent in coronene. This is because sumanene has a dipole moment along the *C*_*3*_ axis, enhancing the intensities of modes involving this plane.^64^ The lowering of the symmetry in the case of the sumanene cation and increasing of the permanent dipole moment by obtaining a more puckered structure are expected to give rise to this intense IR feature. Additionally, as mentioned above, there are two distinct CH_oop_ modes, at 815 and 795 cm^–1^, due to the sp^2^ and sp^3^ hybridized CH bonds in the sumanene cation, which are the characteristic of the peripheral pentagons, whereas the coronene cation has only one band at 848 cm^–1^ due to sp^2^ hybridized C–H bonds (duos). The C–C stretching modes at 1393 and 1437 cm^–1^ for the sumanene cation are observed to be blue-shifted from that of the coronene cation, present at 1330 cm^–1^. Coronene being a very compact PAH, its C–C stretching mode in the gas phase is expected to be intense and broad according to theoretical studies, which is probably caused by the presence of additional unresolved absorptions.^30^ It is expected that very large, asymmetric, and irregular PAHs will have even more infrared activity induced in this wavelength region because of the above-mentioned reason.^65^ In the sumanene cation, the C–C stretching modes are influenced by the presence of three pentagon rings, since the C–C bonds are more strained to adopt the bowl structure and indeed they have higher frequencies than those of sumanene. The CCC skeletal mode observed at 719 cm^–1^ for the sumanene cation is not observed in the case of coronene cation because of its planarity. The overall comparison between the IR spectra of cationic coronene and cationic sumanene provides insight into the effect of planarity on the IR absorptions. The presence of pentagons and strained CC bonds has a significant effect on their IR spectrum.

## 5 Astrophysical Implications

One well-known cooling mechanism of PAHs is vibrational cooling. In the interstellar medium, PAHs are electronically excited by UV photons originating from stellar sources and relax to highly excited vibrational states in their ground electronic state through a radiationless internal conversion. This vibrational energy is then emitted as IR photons, and the internal energy of PAH decreases. This decrease affects the energy at which the next IR photon is emitted. As a result, there is an ever-changing IR emission spectrum during the cooling process. This cascade behavior has a significant effect on the resulting emission spectrum when compared to observations. Hence, the cascade effect on the observational spectra must be considered with theoretical methods.^66,67^ The cascade effect cannot be accounted for easily in experiments with the present technology, except for a few studies that recorded the emission spectra.^62,68−70^ So, caution must be taken in the interpretation of the astronomical spectra based on laboratory spectroscopy.

Infrared absorption spectroscopy is widely used in laboratories for recording the fingerprints of PAH and their derivatives. The experimental gas phase spectra obtained in this work are infrared action spectra of neutral and cationic sumanene, which might differ slightly from the AIBs originating from UV-induced emission. Typically, a small (10–30 cm^–1^) red shift for the emission bands is expected because of anharmonicity.^71,72^ Anharmonic interactions introduce small red shifts in the emission bands when spectator modes are appreciably populated. However, as Mackie et al. demonstrated in their cascade studies, for well-isolated, intense low-frequency bands, such as the CH_oops_ mode, the overall emission spectrum integrated over the cascade will peak at the low-temperature absorption peak. The main effect of the cascade is the red-shaded wing.^61^ For the CH out-of-plane bending modes, it is therefore very appropriate to make a direct comparison between the observed AIB peak positions with the peak position in the measured absorption spectrum. In more congested high-frequency spectral regions, such as the CH stretching or CH-ip/CC stretching regions, the various modes will blend in the cascade, and shifts are apparent. Resonance effects in the CH stretching region may further complicate the comparison.^67^

The 3 μm region, corresponding to the CH stretching modes, is of utmost interest in this study, as sumanene is a unique molecule with both sp^2^ and sp^3^ hybridized carbon sites. The CH stretching mode of sumanene corresponding to the sp^2^ hybridized carbon is observed at 3047 cm^–1^ (3.28 μm) which is slightly blue-shifted from the interstellar AIB at 3.29 μm (Figure 4). The CH symmetric stretching modes of coronene, methylated coronene,^44^ ethylated coronene^45^ at 3065 cm^–1^ (3.26 μm), 3052 cm^–1^ (3.28 μm), and 3051 cm^–1^ (3.28 μm), respectively, are more blue-shifted from the AIB. The CH stretching mode corresponding to the sp^3^ hybridized carbon is observed at 2905 cm^–1^ (3.44 μm). The asymmetric stretching modes due to the sp^2^ bond (antisymmetric with respect to the symmetry operator resulting from the sp^2^ carbon structure) are also observed in the gas phase spectrum of sumanene with a shoulder at 3047 cm^–1^ (3.28 μm), and for the sp^3^ bond with a peak at 2946 cm^–1^ (3.39 μm). The asymmetric stretching of the sp^3^ CH group exactly matches that of the 3.4 μm band of the AIB. It is observed that the curvature of sumanene induced by the pentagons has an influence on the CH stretching modes, which causes a further red shift of the aliphatic modes. Sumanene is a unique PAH with both sp^2^ and sp^3^ hybridized carbon sites with its IR features in the 3–4 μm range matching reasonably well with the AIBs. These spectral signatures certainly point toward the fact that sumanene-like molecules could be present as substructures in the AIB and are astronomically important PAHs to be considered for detection in the ISM. As for coronene, the highly symmetric nature of sumanene results in IR activity in the CH stretching region over a very limited frequency range. Guided by the cascade study on pyrene,^67^ we may surmise that this will result in a single peak in the calculated emission spectrum near the position of the AIB.

As far as sp^3^ vibrational modes are concerned, it is important to compare the vibrational modes of sumanene with those of diamondoids. The CH stretching modes at 3.53 μm, and the CH_2_ (symmetric −3.47 μm, and asymmetric 3.43 μm) stretching modes of the sp^3^ hybridized diamondoid molecules are unique IR emission features, and these have been detected in the emission spectrum associated with the disks around two young stellar objects, HD 97048 and Elias 1.^73^ These two emission bands are basically seen in a very small number of young stellar objects (Herbig AeBe stars, HD 97048 and Elias 1) and do not resemble the PAH AIBs.^74^ A broad absorption band at 3.47 μm has also been attributed to diamond-like molecules^75^ possibly trapped in ice or as part of HAC material.^76^ Moreover, the DFT studies of the vibrational and electronic spectra of these cations show that contributions from the diamondoid cations to the AIB emission bands in the 3.4–3.6 μm range cannot be excluded.^77^ The IR spectrum of sumanene presented in this work demonstrates that its CH stretching motions corresponding to the sp^3^ hybridized CH group are much different from that of diamondoids. The characteristic sp^3^ CH symmetric and asymmetric stretching modes of sumanene are present at 3.44 and 3.39 μm, respectively, implying that the sumanene band positions are blue-shifted from the diamondoid band by 0.03 μm (∼25 cm^–1^) and 0.04 μm (∼34 cm^–1^) respectively. This implies that the spectral signatures of the diamondoids and sumanene in the CH stretching regions are clearly distinguishable and that the origin of the diamondoid features cannot be reassigned to sumanene or likely any of its associated species.

**Figure 7:**
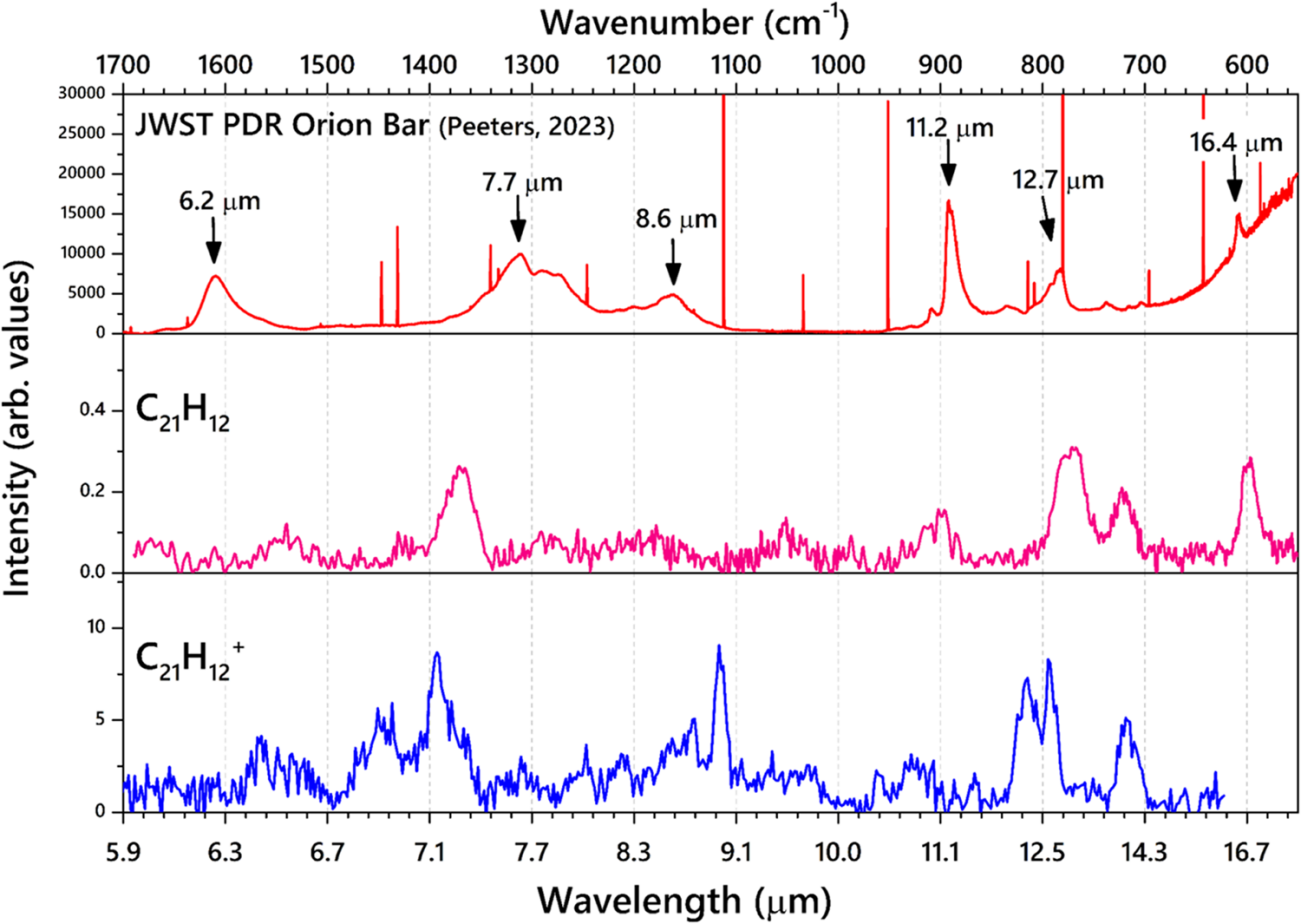
Comparison of the experimental IR spectra of sumanene (C_21_H_12_) and cationic sumanene (C_21_H_12_^+^) with the astronomical AIB from the photodissociation region of the Orion Bar recorded by the JWST. The black arrows point to the PAH features (data obtained from Peeters et al., 2024).^62^

In addition to the 3 μm region, the lower-frequency (600–1700 cm^–1^) region is very interesting to compare with that of the astronomical data. The PAHs demonstrate several active vibrational modes in this region that are indispensable in decoding the AIBs. Figure 7 compares the recorded IR spectra of neutral and cationic sumanene (C_21_H_12_, C_21_H_12_^+^) with the AIBs from the photodissociation region of the Orion Bar observed with the JWST.^30^ The characteristic vibrational features of sumanene and sumanene-like molecules could possibly be present in the AIBs as substructures. It should also be noted that the line width of the experimental spectra is limited by the FELIX laser beam width. Sumanene-like structures are important to study as these molecules bear pentagons in their periphery which induce IR features that are characteristic of the sp^3^ hybridized CH modes. The aliphatic modes arising from the sp^3^ CH modes are thought to correlate with those of the AIBs. As discussed in Sections 4.1 and 4.2, the combination modes along with the sp^3^ hybridized CH motions also give rise to increased IR activity in the mid-IR region. The CH stretching region (Figure 4) demonstrates that aliphatic modes are more like the observational spectra obtained from the structures of the carbonaceous dust in the diffuse ISM, where the 3.4 μm band is expected to have four aliphatic components.^78^

The intense bands from the CCC ring breathing mode and CH_oop_ bending modes of both C_21_H_12_ and C_21_H_12_^+^ exhibit features that are heavily red-shifted from the AIBs. The resemblance is still not satisfactory to assign any specific band unambiguously to that of the AIBs. Moreover, the CH_oop_ bending modes in C_21_H_12_ and C_21_H_12_^+^ contain two distinct features corresponding to the sp^2^ and sp^3^ hybridized carbon bond, which is strikingly different from the observed AIBs. The characteristic CH in-plane bending mode (8.6 μm in the AIB), is observed at 1117 cm^–1^ (9.0 μm) in C_21_H_12_^+^ and as a very weak feature in C_21_H_12_ at 1051 cm^–1^ (9.5 μm). The previous studies have demonstrated that the 8.6 μm band is primarily attributed to the cationic and anionic PAHs, with a particular cation-to-anion ratio.^79,80^ However, those studies mainly focus on the highly symmetric, compact, and planar PAH. The deviation of the CH in-plane peaks from the 8.6 μm peaks could likely be induced due to the puckered structure of the sumanene cation. It has to be remembered that the Ne-tag to sumanene in the IR-PD experiments also induces small peak shifts.^44^

Finally, as already mentioned, we stress that the astronomical AIB spectrum is an emission spectrum, in contrast to the experimental results presented here. Therefore, relative intensities can be influenced by the temperature of the observed source, cascade effect, etc.^71,72,81^ For sumanene, the presence of pentagons is expected to make these molecules very stable, and they are therefore good candidates for the interstellar PAH family. The identification of buckybowls like sumanene and corannulene in the photodissociation regions will be crucial in understanding the relationship between fullerenes and PAHs. In addition, this will also be important to deduce the isomerization process that could take place during the energetic processing in the photodissociation regions to form large and stable PAHs^33^ For instance, the benzenoid PAHs like pyrene are studied to undergo Stone–Wales-like phenomenon to catalyze the formation of pentagons, which is also expected to take place in sumanene upon photodissociation.^82^ When such pentagons are formed by isomerization of a planar PAH, the CC bonds are strained, and the PAH becomes nonplanar. However, sophisticated molecular dynamics simulations are necessary to decipher this process. Experimental^15^ and observational^20^ studies have shown that pentagon formation is inevitable in PAHs. In addition, the smaller PAHs that were detected using the rotational emission spectra hint at the bottom-up growth route for larger PAHs and fullerenes in dense clouds. In that case, it is likely that species containing pentagons are important molecular intermediates in this process, and sumanene is a particularly relevant candidate. So, it seems likely that pentagon-bearing molecules await their ISM detection.

## 6 Conclusions

In this work, the IR spectra of both neutral and cationic sumanene (C_21_H_12_ and C_21_H_12_^+^) were investigated in the 3–17 μm region. Sumanene, being a structural motif with a radius of curvature close to that of C_60_, is astronomically interesting and represents an important class of PAHs for which previous spectroscopic data were largely lacking.

The IR spectrum of neutral sumanene agrees well with the quantum chemically calculated IR spectrum and with the matrix-isolated IR spectrum in the solid *p*-H_2_. The spectrum shows two intense CH out-of-plane bending modes arising from two different sp^2^ and sp^3^ hybridized carbon bands. This is a unique feature absent in any planar PAH that possesses only sp^2^ carbons in its periphery. The CH stretching region is found to contain both the aromatic and aliphatic modes that are also the characteristics of the AIBs, giving insight into the importance of the sp^3^ hybridized carbon periphery in a PAH. However, care must be taken in the interpretation as the experimental spectrum is an absorption, whereas the AIBs represent emission features. Recently, Mackie et al.^69^ used a vibrational anharmonic method to derive infrared emission spectra under interstellar conditions. It was found that the energy cascade inherent in the vibrational relaxation of isolated molecules produced red-shaded wings on their emission profiles, especially in the CH_oop_ bending mode.^64^ Further computational modeling studies are required in this respect. With the aid of computational tools, the emission spectra of sumanene can also be determined as a future scope of this work, as experimental spectra are crucial to understand the AIBs. This will be the first step in determining the emission spectra of buckybowls under interstellar conditions.

The IR spectrum of cationic sumanene attained with the FELion 22-pole ion trap experiments agrees well with the quantum chemically calculated IR spectrum. This study shows that the geometry and the charge state of the PAH crucially affect the IR features, which in turn guides the astronomical modeling studies to gradually pin down the molecules that can be considered further to unravel the AIBs. The influence of the planarity of the PAH on its infrared signatures is also explored. A striking difference of the IR signatures of sumanene with that of coronene was noticed both in the CH stretching region and in the lower-wavenumber regions. In addition to the peripheral carbons, the curvature of sumanene has an influence on the IR fingerprints of the molecule compared to coronene, which was evident in the CC stretching and CCC skeletal modes. This work also provides insight into distinguishing the 3.3 and 3.4 μm features from that of the hydrogenated, methylated, and ethylated PAHs. A detailed discussion about its difference from the 3.4 μm features of the diamondoids is also provided to validate the observational spectrum.

The increase in bowl depth from the planar coronene to corannulene (0.87 Å) and to sumanene (1.11 Å)^83^ provides a prime, systematic approach to understand the influence of curvature induced by pentagons on the IR characteristics of PAHs. Moreover, the excellent correlation of the CH stretching of sumanene with that of the AIB insists that a focused study of species possessing an sp^3^ hybridized periphery is called for. The spectra recorded in this work will also be valuable for NASA’s PAH infrared database.^84^ The IR spectroscopy of this pentagon-bearing PAH, (both C_21_H_12_ and C_21_H_12_^+^), is essential for the detection of sumanene-like species in the molecular clouds.

## Supporting Information Available

Experimental vibrational wavenumbers of C_21_H_12_ compared with the calculated wavenumbers using the B3PW91 6-311++G(2d,2p) method (Table S1); experimental vibrational wavenumbers of C_21_H_12_^+^ compared with the calculated wavenumbers using the B3LYP/N07D method for the anharmonic and B3PW91 6-311++G(2d,2p) method for harmonic vibrations (Table S2); and calculated anharmonic wavenumbers (cm^–1^) and normalized IR intensities (km/mol) of C_21_H_12_^+^ using the B3LYP/N07D method (Table S3) (PDF)
