# Supplementary material for: Infrared Spectroscopy of Neutral and Cationic Sumanene (C21H12 & C21H12+) in the Gas Phase: Implications for Interstellar Aromatic Infrared Bands (AIBs)
Source: ACS Earth Space Chem. 2025 Mar 27;9(4):898–910. doi: 10.1021/acsearthspacechem.4c00393 (PMC12010423; doi:10.1021/acsearthspacechem.4c00393)
Supplement: Supplementary file 1 — sp4c00393_si_001.pdf [file sp4c00393_si_001.pdf]

Infrared spectroscopy of neutral and cationic sumanene ($C_{21}H_{12}$ & $C_{21}H_{12}^{+}$) in the gas phase: Implications for the interstellar Aromatic Infrared Bands (AIBs)

Pavithraa Sundararajan[1], Piero Ferrari[2*], Sandra Brünken[2*], Wybren Jan Buma[3,2], Alessandra Candian[3], Alexander Tielens[1,4]*

[1] Leiden Observatory, Leiden University, Einsteinweg 55, 2333 CC Leiden, The Netherlands.

sundararajan@strw.leidenuniv.nl.

[2] Radboud University, Institute for Molecules and Materials, FELIX Laboratory, Toernooiveld 7, 6525 ED, Nijmegen, The Netherlands.

[3] Anton Pannekoek Institute, University of Amsterdam, Science Park 904, 1098XH, Amsterdam, The Netherlands.

[4]Astronomy Department, University of Maryland, College Park, MD 20742, USA.

Table S-1: Experimental vibrational wavenumbers of $C_{21}H_{12}$ compared with the calculated wavenumbers using the B3PW91 6-311++G(2d,2p) method:

Harmonic[a]		Experimental	
Wavenumber (cm^{-1})	I[b]	Wavenumber (cm^{-1})	I (norm)[c]
128	0		
140	1		

211	8		
280	0		
314	0		
318	1		
414	5		
428	0		
479	2		
491	10	483.1	9.0
641	0		
554	0		
566	3	556	2.8
607	0		
610	23	596.9	13.7
621	0		
641	0		
727	15	722.6	12.2
736	7		
775	0		
779	53	772.4	17.7
794	35	770.6	15.3
795	0		
836	0		
906	0		
919	7	898.1	9.1
932	0		
935	2		
952	0		
1014	1	987.6	3.9
1055	0		
1083	1		
1084	7	1051.2	10.2
1123	0		
1126	0		

1176	0		
1179	2	1178.4	15.5
1211	4		
1245	2		
1255	0		
1318	0		
1332	1	1290.7	9.1
1345	5		
1407	53	1371.2	29.4
1414	2		
1451	0		
1464	6	1430.8	8.1
1481	0		
1574	0	1539.9	6.7
1585	0		
1586	0		
1645	1		
2899	54	2905.4	54.5
2899	0		
2959	4		
2959	13	2946.4	26.5
3031	0		
3031	12	2998.6	14.3
3048	64	3047.8	100.0
3049	9		

[a] Obtained from Weber et. al. 2022.

[b] Normalized intensities with respect to 767.7 cm^{-1} with line intensity 53.3 km/mol.

[c] Normalized intensities with respect to 3047.8 cm^{-1}.

Table S-2: Experimental vibrational wavenumbers of $C_{21}H_{12}^{+}$ compared with the calculated wavenumbers using the B3LYP/N07D method for the anharmonic and B3PW91 6-311++G(2d,2p) method for harmonic vibrations:

Anharmonic		Harmonic[b]		Experimental	
Wavenumber (cm^{-1})	I (norm)[a]	Wavenumber (cm^{-1})	I (norm)[c]	Wavenumber (cm^{-1})	I (norm)[d]
41.4	2.4	32.5	2.2		
130.2	0.1	127.8	0.1		
140.8	1.1	142.3	0.9		
180.6	3.8	180.0	3.5		
211.0	4.1	210.5	3.4		
264.4	0.5	270.1	0.2		
305.7	0.1	306.7	0.1		
308.5	0.2	311.5	0.0		
318.8	0.2	316.2	0.1		
318.9	0.3	328.1	0.6		
398.9	0.0	407.0	0.0		
403.9	0.2	412.8	12.1		
411.4	10.1	428.4	0.1		
450.2	0.2	444.7	0.2		
473.8	2.5	472.3	5.2		
476.8	6.0	477.3	1.1		
499.2	6.1	501.9	8.4		
518.0	0.0	522.3	1.0		
522.6	0.2	531.2	0.0		
565.7	1.9	567.8	1.8		
580.4	4.2	586.9	11.9		
583.2	3.8	589.4	3.6		
617.7	0.1	601.4	0.1		
637.8	0.4	617.7	0.6		
715.5	12.5	638.4	0.8		
724.9	0.8	714.6	21.8	719.7	47.9
727.5	0.0	724.2	1.7		
728.8	0.2	726.7	0.2		
773.3	0.0	730.2	0.2		
786.8	3.8	779.0	0.0		
789.7	18.2	790.5	15.9	795.4	51.4
801.0	0.1	791.7	15.2		

812.0	1.5	802.0	2.9		
815.1	3.8	821.4	20.3	815.9	68.4
848.1	0.1	829.6	4.5		
892.4	0.6	842.9	0.8		
902.1	1.0	900.8	1.7		
918.3	0.5	902.5	1.9	906.3	9.3
922.6	3.6	924.2	4.5		
936.2	0.5	927.0	4.7	929.8	26.3
952.8	0.1	947.5	0.4		
962.4	0.8	960.6	0.6		
962.9	1.8	969.6	0.7		
996.0	0.2	970.3	1.6		
1025.8	1.3	1027.4	1.3		
1042.4	4.0	1039.6	9.4		
1054.4	1.3	1048.5	7.9		
1088.7	1.0	1059.7	1.5	1064.7	18.5
1102.0	12.3	1103.9	2.5		
1112.3	2.4	1113.8	4.7	1094.7	17.9
1118.8	0.3	1118.7	6.0		
1121.2	26.7	1119.4	54.9	1117.8	58.2
1123.0	0.0	1128.3	0.1		
1139.0	0.0	1135.4	1.5		
1181.7	0.0	1146.2	5.1	1140.2	33.1
1182.1	1.0	1183.1	1.0		
1205.2	3.3	1189.3	0.2		
1209.8	1.0	1217.1	6.5	1212.3	30.8
1221.4	0.2	1222.1	0.7		
1253.7	0.7	1245.6	5.3		
1257.8	0.3	1256.5	8.6	1246.6	28.8
1325.2	2.1	1268.2	0.0		
1340.8	0.8	1324.0	1.7		
1341.0	0.0	1348.0	0.0		
1356.5	12.4	1362.0	12.9	1366.3	31.3
1366.4	30.7	1386.9	6.2		

1387.8	66.4	1393.0	10.1		
1396.6	13.2	1399.6	11.0	1393.9	100.0
1401.0	0.8	1401.4	82.3		
1406.0	4.9	1414.1	4.1		
1427.9	54.5	1415.1	0.5		
1448.6	1.2	1436.4	100.0	1437.1	65.8
1452.9	0.8	1461.4	0.4		
1457.5	0.1	1465.3	3.3		
1479.7	100.0	1468.8	8.5	1464.7	21.3
1490.5	0.6	1511.6	0.6		
1515.5	3.5	1539.9	53.4	1565	47.2
1540.3	0.4	1552.4	21.8	1574.7	18.7
1562.4	13.7	1575.7	0.2		
1578.6	0.7	1591.2	1.0		
2902.4	2.3	1626.7	6.6	1654.7	13.8
2914.9	0.1	2982.6	4.8		
2915.2	0.1	2994.5	0.0		
2950.4	0.1	2995.4	0.4		
2964.6	0.9	3040.5	0.0		
2964.8	1.0	3056.2	0.2		
3067.9	0.0	3056.3	0.1		
3068.1	0.4	3127.0	0.4		
3078.2	4.0	3127.8	0.0		
3079.5	1.3	3128.1	0.1		
3081.0	0.2	3139.1	0.3		
41.4	2.4	3142.0	1.3		
130.2	0.1	3142.1	0.2		

[a] Normalized intensities with respect to 1497.7 cm^{-1} with line intensity 206.0 km/mol.

[b] The scaling factors used for the harmonic wavenumbers 0.9807 > 2500 cm^{-1}

[c] Normalized intensities with respect to 1436.4 cm^{-1} with line intensity 245.5 km/mol.

[d] Normalized intensities with respect to 1393.9 cm^{-1}.

Table S-3: Calculated anharmonic wavenumbers (cm^{-1}) and normalized IR intensities (km/mol) of $C_{21}H_{12}^+$ using the B3LYP/N07D method (Note: due to an exhaustive list of 4095 anharmonic frequencies, only the ones that have normalized intensities > 0.5 is presented here):

Wavenumber (cm^{-1})	I (norm)	Wavenumber (cm^{-1})	I (norm)	Wavenumber (cm^{-1})	I (norm)
566.6	7.4	1432.3	0.6	2164.8	1.8
572.5	0.8	1434.9	5.9	2181.7	2.3
710.4	2.2	1435.0	7.2	2183.7	1.0
779.6	1.5	1435.7	45.4	2184.3	1.1
781.9	2.2	1436.1	4.1	2204.9	0.7
782.4	0.7	1436.3	46.3	2207.9	0.9
786.4	0.6	1439.8	10.8	2209.1	1.6
792.3	1.0	1440.4	15.2	2226.3	1.1
793.8	2.1	1442.3	1.2	2236.2	1.5
795.9	1.5	1443.6	117.3	2241.6	1.8
817.2	0.8	1443.9	14.7	2255.3	1.4
819.0	41.7	1452.5	2.7	2255.9	0.7
830.4	5.9	1455.9	0.6	2259.7	0.9
895.2	2.5	1458.8	1.0	2268.2	1.1
897.0	2.1	1459.8	3.4	2272.1	0.7
898.4	0.6	1461.7	1.1	2273.4	1.7
900.6	0.6	1464.4	3.6	2283.7	1.3
922.4	7.4	1466.0	0.8	2287.2	1.5
924.8	1.9	1467.1	0.6	2288.4	0.7
927.3	2.2	1467.1	0.6	2293.6	1.4
957.0	0.8	1467.9	1.1	2304.4	0.9
973.2	0.6	1474.4	1.1	2308.7	0.7
976.8	1.3	1476.0	0.4	2312.3	0.6
998.3	0.7	1477.2	0.8	2312.5	1.2
1002.5	2.6	1479.3	1.4	2317.2	1.4
1021.5	0.9	1483.5	0.4	2325.8	0.9
1030.8	0.9	1487.0	0.4	2333.6	0.6
1032.9	4.7	1492.2	1.8	2335.7	0.9
1035.1	2.0	1499.7	1.8	2339.8	4.5
1044.3	2.4	1508.6	3.8	2343.7	1.3

1044.3	0.9	1512.5	19.6	2353.9	2.2
1048.7	1.5	1513.3	43.0	2356.3	0.7
1056.1	0.7	1516.0	26.5	2370.6	1.1
1091.5	0.7	1516.2	1.0	2373.9	2.3
1099.5	4.9	1517.7	1.2	2381.2	2.5
1100.6	0.7	1518.1	4.2	2392.5	0.8
1101.0	2.1	1518.2	0.9	2392.8	1.1
1106.9	5.2	1523.0	0.8	2395.0	1.5
1110.6	2.8	1524.7	2.1	2398.1	2.0
1114.1	7.6	1525.9	24.4	2411.9	1.1
1114.7	9.1	1526.3	1.4	2423.9	3.3
1120.9	0.7	1527.0	2.9	2428.6	0.6
1128.0	0.9	1527.0	2.7	2435.5	1.0
1129.0	0.6	1527.9	0.6	2447.6	2.2
1135.0	1.5	1529.7	1.6	2449.6	2.3
1139.2	0.7	1530.3	1.1	2458.9	3.3
1140.3	0.9	1531.0	25.8	2467.0	4.1
1143.0	8.2	1531.9	31.4	2468.1	1.2
1144.7	1.7	1534.7	61.1	2471.5	1.1
1148.9	2.7	1535.9	2.7	2476.7	0.7
1152.1	1.0	1537.7	1.6	2478.2	9.3
1188.8	0.6	1538.8	0.6	2492.7	1.0
1191.3	1.2	1542.5	0.6	2496.1	1.5
1191.7	5.3	1543.1	6.2	2551.9	1.1
1209.5	0.5	1543.1	2.2	2556.2	13.4
1213.8	5.6	1545.3	2.7	2561.4	2.9
1221.2	0.7	1545.8	0.6	2579.4	15.4
1224.2	6.9	1546.6	4.2	2584.5	1.8
1227.6	4.4	1548.5	0.7	2605.6	0.7
1230.8	0.5	1550.6	1.0	2611.7	3.5
1231.1	5.5	1551.8	17.4	2630.3	0.6
1233.3	5.0	1553.8	1.3	2637.3	0.9
1241.7	0.6	1556.3	0.8	2643.8	4.8
1249.7	2.9	1557.5	1.3	2665.5	0.9
1252.6	3.7	1558.6	0.6	2675.1	1.2
1255.4	1.1	1585.9	1.0	2685.4	1.1

1258.8	0.6	1590.0	1.7	2693.4	4.5
1263.6	4.8	1594.3	2.0	2696.8	1.3
1301.9	0.7	1602.2	6.4	2715.4	1.9
1309.3	5.8	1605.1	3.9	2718.1	1.7
1322.4	1.5	1605.5	3.3	2742.2	1.7
1335.2	0.7	1608.7	1.6	2752.8	0.6
1344.1	0.9	1615.5	2.1	2754.8	0.1
1345.9	0.6	1621.7	1.0	2757.8	1.3
1347.9	1.3	1626.6	1.6	2762.1	1.8
1355.0	0.8	1632.6	5.5	2774.1	1.4
1357.9	1.9	1646.1	0.6	2776.1	4.4
1360.3	2.9	1656.7	0.6	2785.6	2.7
1361.7	1.2	1658.0	0.7	2791.1	0.6
1361.9	4.9	1667.5	1.1	2793.4	1.5
1365.9	0.5	1700.0	1.7	2794.0	0.6
1371.2	0.9	1706.1	0.9	2794.6	7.4
1372.6	1.3	1714.2	2.8	2804.9	1.3
1372.8	0.9	1722.5	2.8	2819.7	4.8
1375.0	3.5	1725.8	0.8	2826.8	0.6
1375.6	1.9	1732.1	1.7	2827.4	0.7
1380.2	1.5	1733.9	0.6	2848.5	3.4
1382.6	5.0	1798.4	1.1	2850.3	1.7
1390.0	1.4	1802.4	2.2	2870.7	1.0
1390.3	14.1	1807.0	2.7	2880.3	2.1
1391.4	25.0	1820.0	0.7	2881.5	2.7
1393.2	3.0	1839.8	0.7	2914.0	3.4
1393.2	0.6	1845.6	0.6	2918.3	0.7
1393.7	5.8	1857.2	0.6	2933.7	0.8
1394.4	6.5	1861.2	0.7	2940.1	0.6
1394.6	1.1	1865.3	1.4	2944.4	1.8
1395.8	82.4	1877.7	2.8	2946.1	0.8
1397.5	1.5	1884.0	2.4	2965.6	1.5
1398.7	0.6	1897.6	0.9	2969.7	0.9
1399.8	1.9	1900.2	3.7	2980.3	0.6
1401.0	0.9	1900.6	0.6	2981.0	1.5
1401.1	0.7	1914.5	0.6	2995.8	0.9

1402.3	16.2	1937.3	0.9	3012.6	0.6
1404.5	5.4	1970.7	0.8	3013.5	1.3
1404.8	0.5	1990.3	1.2	3034.1	1.0
1405.7	29.4	2014.8	5.8	3045.9	2.2
1406.7	3.3	2027.0	0.8	3059.3	3.6
1412.8	1.0	2034.8	1.5	3068.8	2.6
1416.3	0.7	2042.8	0.5	3113.6	0.9
1417.8	6.7	2056.7	2.5	3118.6	0.6
1420.6	0.6	2071.0	0.9	3134.5	2.2
1421.6	1.4	2076.6	0.8	3155.4	1.0
1421.9	0.8	2077.4	3.0	5703.7	0.7
1425.9	2.8	2079.2	1.7	5731.2	0.6
1426.0	0.6	2099.7	0.5	5828.2	0.6
1428.9	6.3	2110.2	1.1	6068.7	0.8
1429.3	1.8	2118.5	1.1	6068.8	1.1
1429.9	0.7	2141.8	1.1	6078.8	0.9
1430.7	1.1	2143.2	1.2		
1432.0	8.3	2161.1	0.9		
